# Soluble epoxide hydrolase modulates immune responses in activated astrocytes involving regulation of STAT3 activity

**DOI:** 10.1186/s12974-019-1508-2

**Published:** 2019-06-08

**Authors:** Chia-Chi Hung, Yi-Hsuan Lee, Yi-Min Kuo, Pei-Chien Hsu, Huey-Jen Tsay, Ying-Ting Hsu, Chih-Chin Lee, Jia-Jun Liang, Feng-Shiun Shie

**Affiliations:** 1 Institute of Physiology, Brain Research Center, National Yang-Ming University, Taipei, Taiwan, Republic of China; 2 Institute of Neuroscience, Brain Research Center, National Yang-Ming University, Taipei, Taiwan, Republic of China; 3 Center for Neuropsychiatric Research, National Health Research Institutes, No.35 Keyan Road, Zhunan Town, Miaoli County, 350 Taiwan, Republic of China

**Keywords:** Astrocyte activation, Soluble epoxide hydrolase, Immune responses, GFAP, STAT3

## Abstract

**Background:**

Astrocyte activation is a common pathological feature in many brain diseases with neuroinflammation, and revealing the underlying mechanisms might shed light on the regulatory processes of the diseases. Recently, soluble epoxide hydrolase (sEH) has been proposed to affect neuroinflammation in brain injuries. However, the roles of astrocytic sEH in brains with neurodegeneration remain unclear.

**Methods:**

The expression of astrocytic sEH in the brains of APPswe/PSEN1dE9 (APP/PS1) mice developing Alzheimer’s disease (AD)-like pathology was evaluated by confocal imaging. LPS-activated primary astrocytes with mRNA silencing or overexpression of sEH were used to investigate its regulatory roles in astrocyte activation and the induction of pro-inflammatory markers. Primary astrocytes isolated from a sEH knockout (sEH^−/−^) background were also applied.

**Results:**

The immunoreactivity of sEH was increased in activated astrocytes in parallel with the progression of AD in APP/PS1 mice. Our data from primary astrocyte cultures further demonstrate that the overexpression of sEH ameliorated, while the silencing of sEH mRNA enhanced, the lipopolysaccharides (LPS)-induced expression of pro-inflammatory markers, such as inducible nitric oxide, cyclooxygenase 2 (COX-2), and pro-inflammatory cytokines. These findings suggest that sEH negatively regulates astrocyte immune responses. Enhanced immune responses found in LPS-activated sEH^−/−^ astrocytes also support the notion that the expression of sEH could suppress the immune responses during astrocyte activation. Similarly, sEH^−/−^ mice that received intraperitoneal injection of LPS showed exacerbated astrocyte activation in the brain, as observed by the elevated expression of glial fibrillary acidic protein (GFAP) and pro-inflammatory markers. Moreover, our data show that the phosphorylation of the signal transducer and activator of transcription 3 (STAT3) was upregulated in activated astrocytes from sEH mouse brains, and the pharmacological blockade of STAT3 activity alleviated the pro-inflammatory effects of sEH deletion in LPS-activated primary astrocytes.

**Conclusions:**

Our results provide evidence, for the first time, showing that sEH negatively regulates astrocytic immune responses and GFAP expression, while the underlying mechanism at least partly involves the downregulation of STAT3 phosphorylation. The discovery of a novel function for sEH in the negative control of astrocytic immune responses involving STAT3 activation confers further insights into the regulatory machinery of astrocyte activation during the development of neurodegeneration.

**Electronic supplementary material:**

The online version of this article (10.1186/s12974-019-1508-2) contains supplementary material, which is available to authorized users.

## Introduction

Astrocytes are major glial cells residing in the brain. Astrocytes become activated during the pathogenesis of many neurodegenerative diseases such as Alzheimer’s disease (AD). In AD, activated astrocytes are in proximity to β-amyloid (Aβ) plaques, one of the pathological hallmarks of AD, which have been implicated in the progression of AD [1, 2]. A plethora of effectors derived from activated astrocytes are involved in various physiological functions, including neuronal migration, synaptogenesis, and neuroplasticity, as well as in many pathological conditions [3–5]. Conjointly with the impacts derived from microglial activation, the uncontrolled release of these effectors from activated astrocytes causes neuroinflammation and instigates oxidative stress in the diseased brain [6]. Oxidative stress increases the expression of pro-inflammatory genes, which leads to the increased production of interleukin-6 (IL-6), tumor necrosis factor α (TNFα), and prostaglandins, through cyclooxygenase 2 (COX2), and nitric oxide (NO) through the activity of inducible NO synthase (iNOS) [7–9]. The resulting consequences, in turn, release more unwanted effectors that repeatedly propagate oxidative stress, ultimately leading to neurotoxicity. Among the many signaling pathways involved in the induction of pro-inflammatory genes, the phosphorylation of the signal transducer and activator of transcription 3 (STAT3) was recently reported to be critical for the induction of glial fibrillary acidic protein (GFAP) and pro-inflammatory genes such as TNFα and IL-6 [10–12]. These results suggest that STAT3 may play an important role in the control of astrocyte activation and the associated immune responses.

Glia-mediated neuro-inflammation and subsequent neurodegeneration are common pathological features shared by many neurodegenerative diseases, and the inflammatory responses derived from activated astrocytes exacerbate the pathogenesis of these diseases [13]. However, compelling evidence shows that astrocyte activation can also be beneficial for disease recovery and survival [14, 15]. Indeed, the ablation of activated astrocytes disrupts glial scar formation, leading to the persistent infiltration of inflammatory cells and the failure to recover the integrity of the blood brain barrier [16, 17]. In AD, it has been shown that activated astrocytes mediate Aβ degradation, suggesting that they may function against Aβ accumulation [18–20]. These findings suggest that astrocyte activation is a double-edged sword that can be both beneficial and detrimental to neuronal functions [21], thus, revealing the regulatory mechanism of astrocyte activation may lead to a better understanding of neuronal survival and death in the diseased brain. The fine-tuning of astrocyte activation that enhances neuronal survival and ablates the self-reinforcing cycles of neuro-inflammation is critical for developing treatments for diseased brain with robust neuro-inflammation [22].

Soluble epoxide hydrolase (sEH) has recently gained increasing attention because the inhibition of hydrolase activity by sEH can result in sustained levels of epoxyeicosatrienoic acids (EETs) and provide neuroprotection in cardiovascular disease and brain injuries [23–28]. Epoxygenases, a subgroup of enzymes in the cytochrome P450 family, metabolize arachidonic acid into hydroxyeicosatetraenoic acids (HETEs) and EETs [29], the latter of which can be further metabolized into less active forms of DiHETEs by sEH, which is encoded by the gene Ephx2. The C-terminus of sEH is the site of the epoxide hydrolysis activity responsible for producing DiHETEs, while the N-terminal domain of sEH possesses phosphatase activity, with lysophosphatidic acid as its endogenous substrate [30]. sEH is primarily found in the liver, the kidney, the cardiovascular system, and the brain [31, 32]. The expression level of sEH was increased in mice with pilocarpine-induced epilepsy, while the genetic knockout of sEH in mice made them more susceptible to inducible seizures [33]. However, the mechanism through which astrocytic sEH regulates glia-mediated neuroinflammation remains unclear. In this study, we demonstrate that the increased expression of sEH in activated astrocytes in the vicinity of Aβ plaques was associated with the progression of AD in a mouse model. The immune regulatory roles of astrocytic sEH in LPS-activated astrocytes were investigated using in vitro and in vivo models.

## Materials and methods

### Materials

Lipopolysaccharide (LPS, *Escherichia coli* O55:B5) was purchased from Calbiochem. Antibodies for inducible nitric oxide synthase (iNOS) and β-actin were purchased from BD Transduction Lab and Novus Biologicals, respectively. Cyclooxygenase-2 (COX-2) and glial fibrillary acidic protein (GFAP) were purchased from Abcam. Total STAT3 and phospho-STAT3 (p-STAT3) were purchased from Cell Signaling Technology. ELISA kits for TNFα and IL-6 were purchased from Invitrogen. 12-(3-adamantan-1-yl-ureido)-dodecanoic acid (AUDA) and N-acetyl-S-farnesyl-L-cysteine (AFC) were purchased from Sigma-Aldrich. Poly-ornithine was purchased from BD Biosciences. Papain and DNase I were purchased from Worthington Biochemical. Fetal bovine serum was purchased from HyClone and heat-inactivated. Culture media and penicillin/streptomycin were purchased from Gibco, and other common chemicals were from Sigma-Aldrich, unless stated otherwise.

### Animals

Breeding pairs of wild-type (Wt) controls and APPswe/PSEN1dE9 (APP/PS1) transgenic mice, overexpressing both human APP695 Swedish mutations (HuAPP695swe) and a mutant human presenilin 1 (PS1-dE9), were originally obtained from the Jackson laboratory. The transgenic genotypes of both APP and PS1 were detected using PCR, according to the manufacturer’s instruction. APP/PS1 transgenic mice that were heterozygous for the transgenes and Wt littermates were used as controls. Given that LPS has been widely used to induce inflammatory responses [9, 12, 22], a single dose of LPS was administered intraperitoneally to mice at 5 mg/kg body weight. Wt and sEH^−/−^ mice that received a single intraperitoneal injection of saline were served as sham control. Forty-eight hours after LPS injection, brain tissues were harvested for later experiments. For primary astrocytic cultures, Wt mice were purchased from the National Laboratory Animal Center (Taipei, Taiwan), and sEH^−/−^ mice (C57BL/6/B6.129X-*Ephx2*^*tm1Gonz*^/J) were generously provided by Dr. Tzong-Shyuan Lee. Mice were maintained at the NHRI Laboratory Animal Center with free access to food and water. All experiments were performed as approved by the NHRI IACUC.

### Primary astrocytic cultures

Primary astrocytic cultures were derived from the cortices of P1 to P3 neonates. Cells were dissociated using an enzyme solution, containing Dulbecco’s modified Eagle’s medium (DMEM), ethylenediamine tetraacetic acid (0.5 mmol/L), L-cysteine (0.2 mg/ml), papain (15 U/ml), and DNase I (200 μg/ml), followed by trituration. Culture medium (DMEM with 10% fetal bovine serum, 100 U/ml penicillin, and 100 μg/ml streptomycin) was changed after 24 hours of initial seeding. To obtain enriched primary astrocytes, the astrocytic monolayer was repeatedly trypsinized (at 90% confluence) and reseeded twice. On the 14th day in vitro (14 DIV), primary astrocytes were reseeded in poly-ornithine-coated 24-well culture plates for experiments, and a fraction of the cells was cultivated on chambered slides at 1 × 10^5^ cells per well, followed by GFAP staining, to determine the purity of the astrocytes. The purity of primary astrocyte cultures was approximately 95%. Primary cultures were treated with 10 ng LPS/ml of culture medium for 24 h. To inhibit STAT3 phosphorylation in LPS-activated astrocytes, 1-h pretreatment with 10 μM of the STAT3 inhibitor, stattic (Abcam), was applied.

### Genetic manipulations of sEH by using siRNA and over-expression plasmid

Small interfering RNA duplexes targeting sEH (si-sEH), consisting of a pool of 3 target-specific siRNAs designed to knock down sEH gene expression, and a scramble control were purchased from Santa Cruz Biotechnology, and an Ephx2 mouse cDNA clone for the overexpression of sEH and a vehicle control were purchased from OriGene. Primary cell cultures were transfected with siRNAs, plasmid, or controls at a final concentration of 30 nM in serum-free Opti-MEM by using the TransIT®-siQUEST™ transfection reagents (Mirus) for 48 h, followed by 24-h treatments of LPS. Quantification of sEH gene expression was evaluated by quantitative polymerase chain reaction (qPCR), as described below.

### RNA extraction, reverse-transcription, and qPCR

Total RNA was extracted with an RNeasy Plus Mini Kit (Qiagen) according to the manufacturer’s instructions. RNA (1 μg) was reverse transcribed into cDNA using a random primer and a SuperScript III Reverse Transcriptase Kit (Invitrogen). Quantitative PCR was conducted using Luminaris Color HiGreen qPCR Master Mix (Thermo) and an ABI PRISM 7500 Real-Time PCR System. Forward and reverse primer sets for each cDNA were used as follows: 5′-TGGTGGTGACAAGCACATTT-3′ and 5′-AAGGCCAAACACAGCATACC-3′ (for Nos2, NM_010927.3); 5′-GGCCATGGAGTGGACTTAAA-3′ and 5′-CACCTCTCCACCAATGACCT-3′ (for COX-2, NM_011198.3); 5′-GAGGGACAACTTTGCACAGG-3′ and 5′-TCCTGTCTATACGCAGCCAG-3′ (for GFAP, NM_001131020.1); 5′-ATCTCATACCAGGAGAAAGTCAACCT-3′ and 5′-TGGGCTCATACCAGGGTTTG-3′ (for TNF-α, NM_013693.3); 5′-GACCAAGACCATCCAATTCATCTT-3′ and 5′-GGAATGTCCACAAACTGATATGCT-3′ (for IL-6, NM_031168.1); 5′-TGGTGTGGAACATGGCTCTCT-3′ and 5′-ACTGGGATAGATCGGATAACTTTCA-3′ (for sEH, NM_007940.4); and 5′-TGTGTCCGTCGTGGATCTGA-3′ and 5′-GATGCCTGCTTCACCACCTT-3′ (for GAPDH, NM_008084.3). The average cycle threshold (Ct) value was normalized using the GAPDH signal. Relative transcript levels were calculated as *x* = 2^−ΔCt^, in which ΔCt = Ct_target gene_ − Ct_GADH_. For cell cultures, each experimental condition was collected from at least 3 independent cultures.

### Confocal imaging

Confocal microscopy was used for semi-quantification and qualitative analysis. Brain samples were subjected to paraformaldehyde (4% in PBS) fixation, overnight, followed by cryoprotection with sucrose (30% in PBS). Cryosections, at 30 μm thick, were subjected to immunohistochemical analysis using antibodies against Aβ (6E10, 1:200, Biolegend), GFAP (astrocyte marker, 1:200, Abcam), p-STAT3 (1:200, Cell Signaling Technology), or sEH (1:100) for overnight incubation at 4 °C. The corresponding secondary antibodies conjugated with Alexa Fluor (Invitrogen) as indicated in the results were applied for 2 h. Tissues were coverslipped with mounting medium (Vector Lab) containing 4,6-diamiino-2-phenylindole (DAPI) for nuclei counterstaining. Images were acquired using a Leica confocal microscopy imaging system. GFAP immunoreactivity has been widely used for the evaluation of morphological alterations in activated astrocytes in vivo and thus the quantification of GFAP immunoreactivity was used as a measure of astrocyte activation in this study. Semi-quantification of the immunoreactivity from two sections per mouse was performed using MetaMorph imaging software.

### Measurements of sEH activity

Homogenates of the hippocampus or cortex were subjected to measurements of sEH activity, using an epoxide hydrolase activity assay kit (Cayman Chemistry) according to the manufacturer’s instructions. Briefly, the assay utilized Expox Fluor 7, a sensitive fluorescent substrate for sEH that can be used to monitor the activity of sEH. Hydrolysis of the substrate epoxide yielded a highly fluorescent product, 6-methoxy-2-naphthaldehyde, that was monitored at excitation and emission wavelengths of 330 and 465 nm, respectively, using a microplate reader. sEH activity was presented as pmol/min per mg of protein.

### Western blot

The expression levels of COX-2, iNOS, GFAP, total STAT3, p-STAT3, and β-actin were determined using cell lysates or brain homogenates. Samples were lysed in lysis buffer (50 mM Tris, pH 7.4, 150 mM NaCl, 0.5 % sodium dodecyl sulfate and protease inhibitor cocktail) and were subjected to electrophoresis, followed by protein transfer onto PVDF membranes and Western blot analyses using antibodies against COX-2 (1:750), iNOS (1:750), GFAP (1:1000), total STAT3 (1:1000), p-STAT3 (1:1000), and β-actin (1:5000). Targets were detected by the incubation of HRP-labeled secondary antibodies. The corresponding bands, revealed by electrochemiluminescence (ECL) reaction, were analyzed using ImageJ.

### Measurements of cytokines

The culture media from primary astrocytic cultures was subjected to measurements of pro-inflammatory cytokines, including IL-6 and TNFα, using ELISA kits. The assay was performed according to the manufacturers’ instructions. Detection of the results was performed using an ELISA plate reader (SpectraMaxM2, Molecular Devices) at a wavelength of 450 nm.

### Statistical analysis

A two-tailed independent Student’s *t* test was used to test significance. For ANOVA, significance for post hoc multiple comparisons between groups was determined with the Bonferroni test using GraphPad Prism software. Data are presented as the mean ± SEM. Statistical significance was set at *p* < 0.05.

## Results

### Immunoreactivity of sEH in activated astrocytes increased in parallel with the progression of AD

**Fig. 1:**
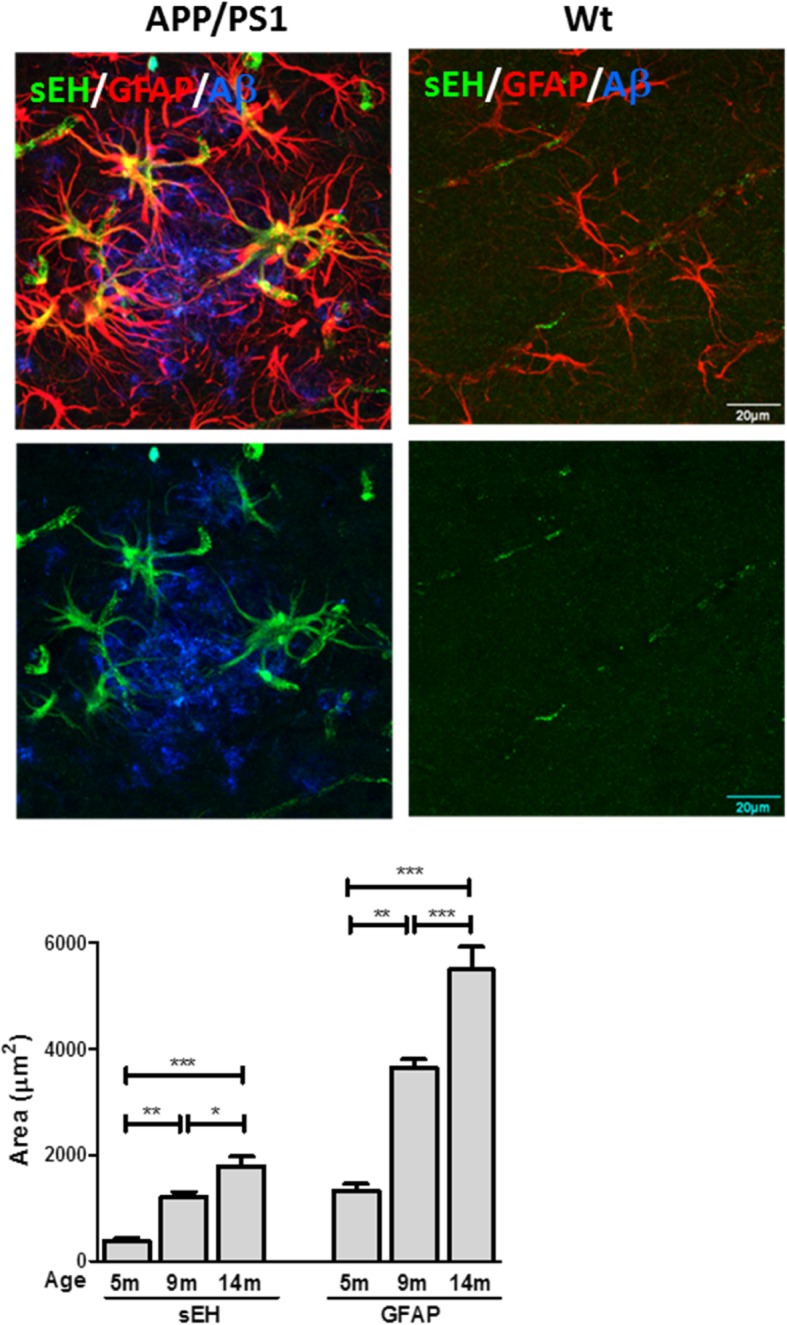
Increased immunoreactivity of sEH in activated astrocytes occurred in parallel with the progression of AD. Representative confocal images show that sEH immunoreactivity (green) was found within activated astrocytes (red) in the vicinity of Aβ plaques (blue) in APP/PS1 mice at the age of 14 months, whereas GFAP-positive astrocytes with less activated morphology showed minimal immunoreactivity for sEH. In age-matched Wt littermates, GFAP-positive astrocytes were observed with resting morphology and contained little immunoreactivity for sEH. In the right panel, the semi-quantification of the total intensity of sEH immunoreactivity centered by Aβ plaques with a diameter of 200 μm in APP/PS1 mice at 5 months (5m, *n* = 5), 9 months (9m, *n* = 10), and 14 months (14m, *n* = 11) of age is shown. The data indicate that the levels of sEH immunoreactivity escalated with aging and in parallel with the progression of astrocyte activation in APP/PS1 transgenic mice. Data are presented as the mean ± SEM. One-way ANOVA and Bonferroni multiple comparison test were performed. *p < 0.05, **p < 0.01, ***p < 0.001

Because activated astrocytes are closely associated with the progression of AD, we first evaluated the expression levels of sEH in activated astrocytes in the vicinity of Aβ plaques. Brain tissues from APP/PS1 transgenic mice at different ages, ranging from 5 to 14 months, were used for fluorescent immunohistochemistry (Fig. 1). Because GFAP has been widely used as a marker for astrocyte activation [2], activated astrocytes were identified by immunohistochemistry with anti-GFAP antibody. The results from confocal imaging show that activated astrocytes surrounding Aβ plaques contained very high immunoreactivity for sEH as shown in 14-month-old APP/PS1 transgenic mice (Fig. 1, upper panel). However, a few astrocytes in the vicinity of Aβ plaques exerting less activated morphology contained little immunoreactivity for sEH. Similarly, GFAP-positive astrocytes with resting morphology in age-matched wild-type (Wt) littermates were negative for sEH. Semi-quantification of the total intensity of sEH immunoreactivity within clusters of GFAP-positive, activated astrocytes, centered by Aβ plaques with a diameter of 200 μm, was performed (Fig. 1, lower panel). The data indicate that the levels of sEH immunoreactivity in the vicinity of Aβ plaques escalated with aging and in parallel with the progression of astrocyte activation in APP/PS1 transgenic mice. As the number of activated astrocytes increased in APP/PS1 transgenic mice compared to the age-matched Wt littermates, the levels of sEH immunoreactivity also increased. These data suggest that the extent of sEH immunoreactivity may be related to the activation state of astrocytes and exposure to Aβ may not necessarily trigger the expression of sEH.

### Expression of sEH in primary astrocytes regulated the LPS-induced immune response

**Fig. 2:**
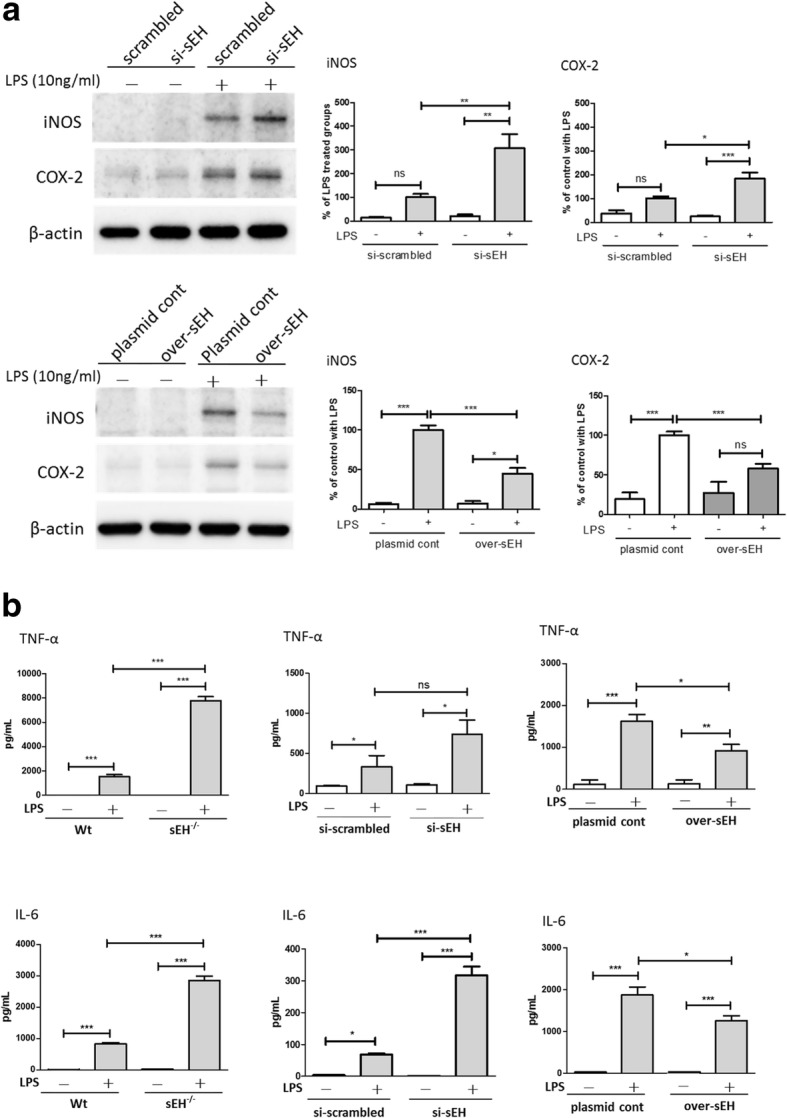
Expression of astrocytic sEH regulated the LPS-induced expression of pro-inflammatory markers. The LPS-induced protein expression levels of pro-inflammatory markers in si-sEH primary astrocytes (*n* = 9), including iNOS (*p* < 0.01) and COX-2 (*p* < 0.05), were significantly increased compared to those in the controls (*n* = 9), while over-sEH astrocytes (*n* = 8) showed a reduction in LPS-induced expression levels of iNOS (*p* < 0.001) and COX-2 (*p* < 0.001) (**a**). Similarly, the secretion of LPS-induced TNFα and IL-6 from primary astrocytes derived from sEH^−/−^ mice (*n* = 12) was significantly increased (*p* < 0.001 for both) compared to the LPS-treated controls (*n* = 10), while the secretion of TNFα and IL-6 from over-sEH astrocytes (*n* = 4) was reduced (*p* < 0.05 for both) compared to the LPS-treated controls (*n* = 4) (**b**). In si-sEH astrocytes (*n* = 4), IL-6 secretion was significantly increased (*p* < 0.001) compared to the LPS-treated controls (*n* = 4), while TNFα was slightly increased compared to the LPS-treated controls. Changes in the expression level of sEH had no effects on the basal immunity of astrocytes without the challenge of LPS. Data are presented as the mean ± SEM. Statistical analysis was performed using one-way ANOVA and Bonferroni multiple comparison test. *p < 0.05, **p < 0.01, ***p < 0.001

To reveal the possible role of astrocytic sEH observed in the activated astrocytes in vivo, we established in vitro models, using LPS to activated primary astrocytes, and then examined the effects of sEH expression on the regulation of astrocyte activation using genetic manipulations. The expression levels of sEH in primary astrocytes derived from wt mice were genetically manipulated by the RNA silencing of sEH (si-sEH) and the overexpression of sEH (over-sEH). The levels of sEH mRNA in si-sEH and over-sEH astrocytes were 28 ± 3% and 5.8 ± 1.2 × 10^6^% of the controls, respectively, as measured by qPCR (Additional file 1: Figure S1A, B). Unexpectedly, the mRNA levels of sEH in si-scrambled control astrocytes and non-transfected primary astrocytes appeared to be downregulated by LPS treatments (Additional file 1: Figure S1C). However, sEH activity was slightly reduced in primary astrocytes treated with LPS (79 ± 4 pmol/min/mg, *n* = 6) compared to non-treated control (86 ± 6 pmol/min/mg, *n* = 6)*.* After genetic manipulation of sEH for 24 h, data show that the expression levels of sEH did not affect the immunity of astrocytes at the basal condition (Fig. 2), while the LPS-induced protein expression levels of pro-inflammatory markers were significantly increased si-sEH astrocytes compared to those in the controls. As indicated in Fig. 2a, LPS-induced expression levels of iNOS and COX-2 in si-sEH astrocytes as measured by Western blot were 307 ± 59% (*p* < 0.01) and 183 ± 25% (*p* < 0.05) of the LPS-treated controls, respectively. In contrast, over-sEH astrocytes showed a reduction in LPS-induced expression levels of iNOS (45 ± 7% of the LPS-treated controls, *p* < 0.001) and COX-2 (58 ± 6% of the LPS-treated controls, *p* < 0.001). Although levels of iNOS and COX-2 were not affected in astrocytes derived from sEH knockout (sEH^−/−^) mice (Additional file 1: Figure S2), the secretion of LPS-induced TNFα (7773 ±333 pg/ml, *p* < 0.001) and IL-6 (2853 ± 141 pg/ml, *p* < 0.001) in sEH^−/−^ astrocytes as measured by ELISA was significantly increased compared to the LPS-treated controls (1523 ± 168 pg/ml and 828 ± 44 pg/ml for TNFα and IL6, respectively). TNFα (918 ± 157 pg/ml, *p* < 0.05) and IL-6 (1268 ± 115 pg/ml, *p* < 0.05) secretion in over-sEH astrocytes was reduced compared to the LPS-treated controls (1620 ± 164 pg/ml and 1876 ± 195 pg/ml for TNFα and IL6, respectively) (Fig. 2b). In si-sEH astrocytes, IL-6 (317 ± 27 pg/ml, *p* < 0.001) secretion was significantly increased compared to the LPS-treated controls (68 ± 4 pg/ml), while TNFα (740 ± 176 pg/ml) was slightly increased compared to the LPS-treated controls (332 ± 141 pg/ml).

**Fig. 3:**
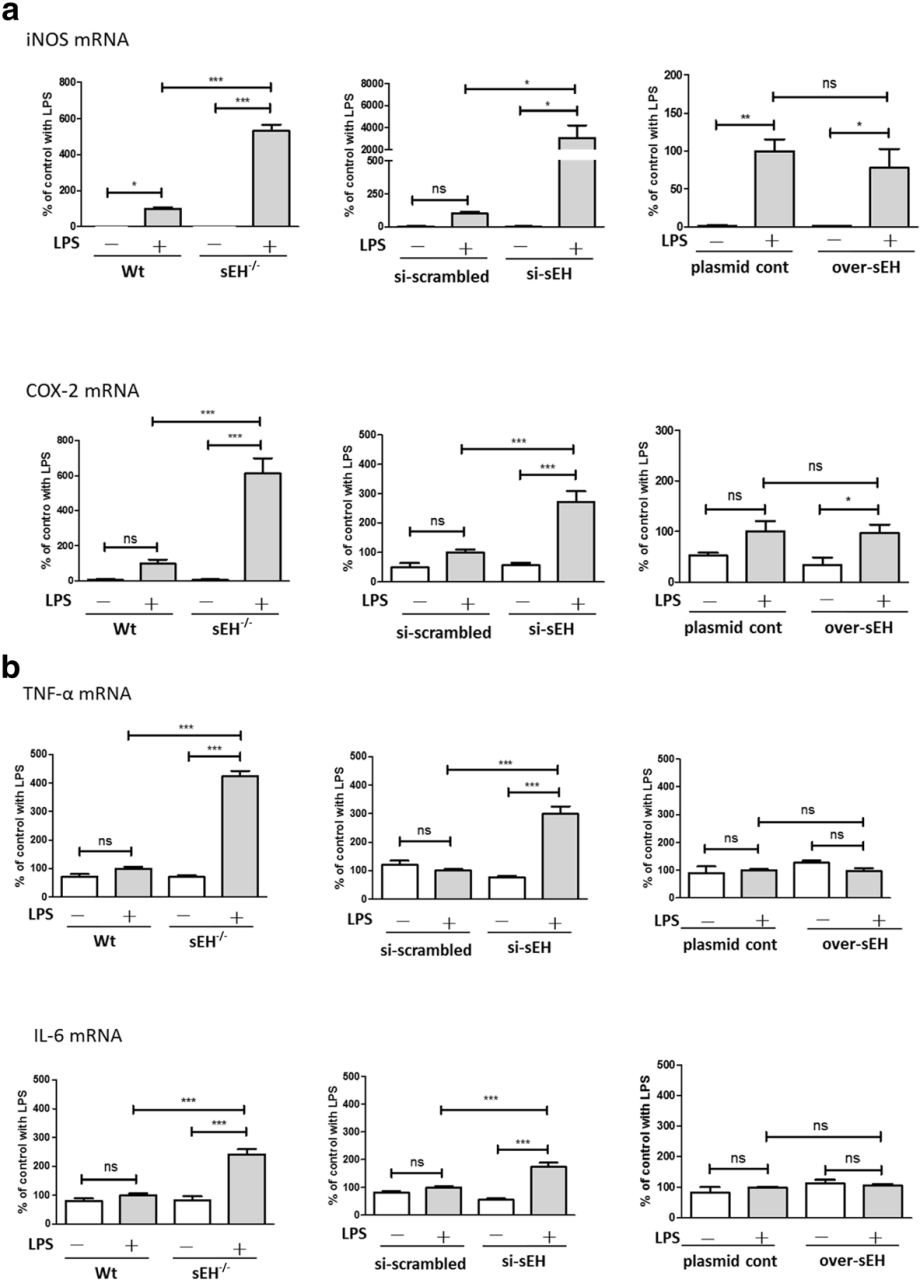
The effects of sEH expression in primary astrocytes on the mRNA levels of LPS-induced pro-inflammatory markers. LPS activated primary astrocytes, as evidenced by significant increases in the mRNA levels of iNOS and COX-2 in Wt, si-scrambled, and plasmid controls (**a**). Manipulations of astrocytic sEH levels using si-sEH (*n* = 9) or sEH^−/−^ (*n* = 6) led to further increases in iNOS (*p* < 0.05 for si-sEH and *p* < 0.001 for sEH^−/−^) and COX-2 (*p* < 0.001 for both) mRNA levels, but the mRNA levels were slightly reduced in over-sEH (*n* = 5). Similarly, the mRNA levels of TNFα and IL-6 in primary astrocytes with either si-sEH or sEH^−/−^ were increased (*p* < 0.001 for both) compared to respective controls, and over-sEH had no effects on the mRNA levels (**b**). Data are presented as the mean ± SEM. One-way ANOVA and Bonferroni multiple comparison test were performed. *p < 0.05; **p < 0.01; ***p < 0.001

The effects of sEH expression on the mRNA levels of the pro-inflammatory markers were then evaluated by qPCR. Similar to that observed at protein levels, the mRNA levels of the pro-inflammatory markers, including iNOS, COX-2, IL-6, and TNFα, were higher in si-sEH or sEH^−/−^ primary astrocytes than those in the respective controls as shown in Fig. 3 a and b. However, the mRNA levels of the pro-inflammatory markers were slightly reduced in over-sEH astrocytes. Of note, LPS treatment increased the mRNA levels of iNOS and COX-2 in the controls, but the mRNA levels of IL-6 and TNFα were not changed. The mRNA levels of pro-inflammatory cytokines induced by LPS in primary astrocytes were only slightly increased, probably due to the use of a low dose of LPS at 10 ng/ml. However, in primary astrocyte cultures, the secretion of IL-6 and TNFα induced by low dose of LPS continued to accumulate, resulting in a significant increase of cytokine secretion as shown in Fig. 2b. These data suggest that astrocytic sEH could act as a suppressor in the regulation of the LPS-activated immune response at both the protein and mRNA levels, and the absence of astrocytic sEH could escalate the immune response. However, the overexpression of astrocytic sEH appears to attenuate the pro-inflammatory markers only at the protein level. Thus, gene expression may not contribute to the anti-inflammatory effects of sEH overexpression, and the roles of sEH in the regulation of astrocyte activation at either the mRNA or protein level might be involved in multiple regulatory pathways.

### The LPS-induced astrocyte activation was exacerbated in the brains of sEH^−/−^ mice

**Fig. 4:**
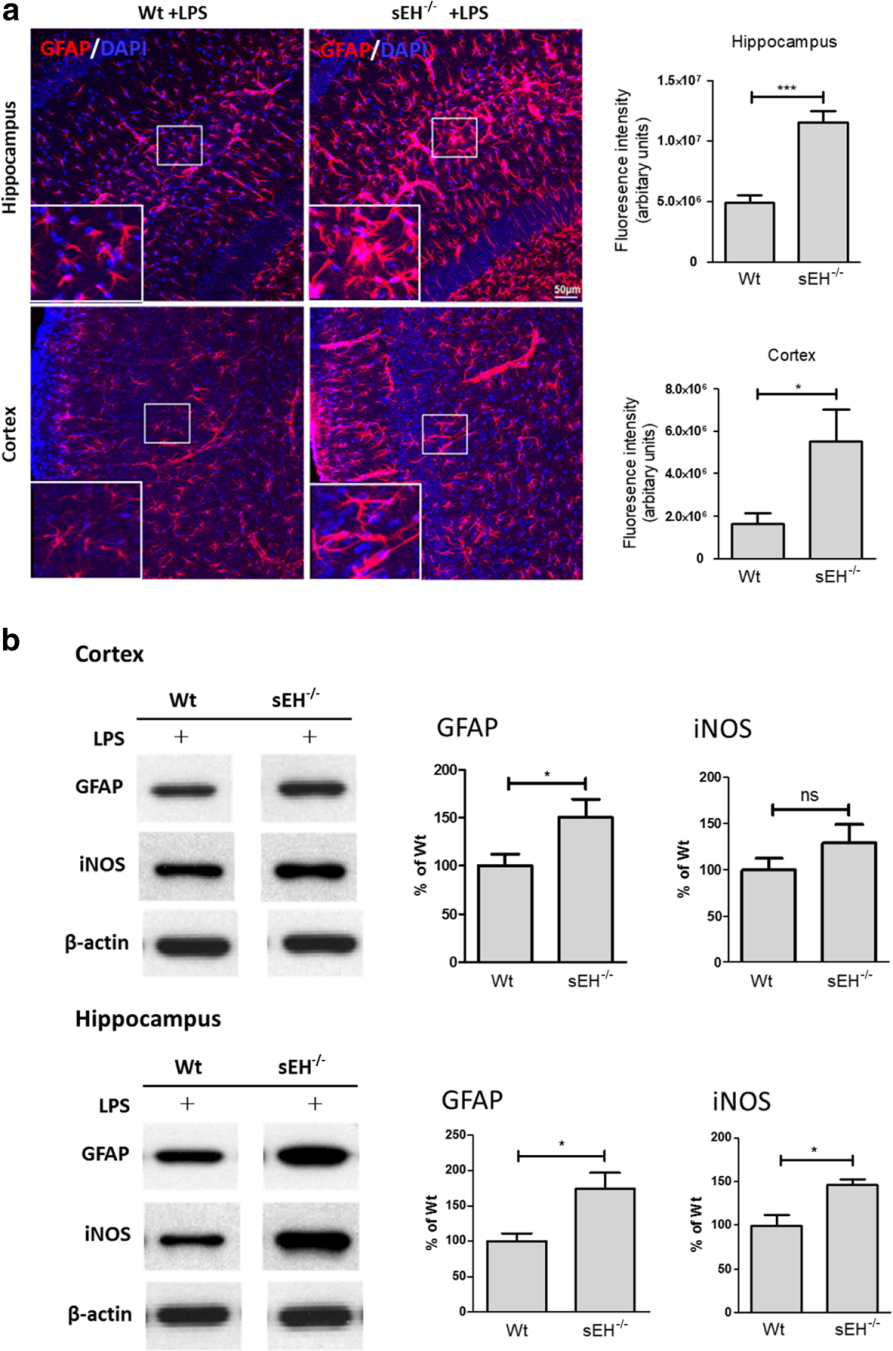
Astrocyte activation was exacerbated in the brains of sEH^−/−^ mice treated with LPS. Confocal imaging shows that astrocyte activation was exacerbated in sEH^−/−^ mice that received intraperitoneal injections of LPS compared to Wt counterparts, as evidenced by the enlarged morphology of GFAP-positive astrocytes in both the cortex and hippocampus (**a**). A magnified image of the boxed area is presented at the lower left of each image for a closer look of GFAP immunoreactivity. This notion is supported by the semi-quantification data, showing that the total intensity of GFAP immunoreactivity is increased in sEH^−/−^ mice with LPS (*n* = 5, *p* < 0.05 for cortex and *p* < 0.001 for hippocampus) compared to Wt counterparts (*n* = 6). Similarly, Western blot analysis also shows increased protein expressions of GFAP and iNOS in both the cortex and hippocampus of sEH^−/−^ mice that received intraperitoneal injections of LPS compared to Wt counterparts, although changes of cortical iNOS were not significant (**b**). Representative blot images for Wt or sEH^−/−^ were obtained from the same blot membrane. Data are presented as the mean ± SEM. Statistical analysis was performed using Student’s *t* test and significance is indicated. *p < 0.05; **p < 0.01

The effect of sEH in the negative regulation of astrocyte activation was further tested using animal models, with cerebral inflammation induced by the peripheral delivery of LPS. Our data show that intraperitoneal injections of LPS (5 mg/kg body weight) in sEH^−/−^ mice exacerbated astrocyte activation in the brain as evidenced by the enhanced reactive morphology of GFAP-positive astrocytes at 48 h after the treatments (Fig. 4a). By Western blot analysis, the protein expressions of GFAP in the cortex (150 ± 19% of Wt control with LPS, *p* < 0.05) and hippocampus (174 ± 22% of Wt control with LPS, *p* < 0.05) were increased in LPS-treated sEH^−/−^ mice compared to those in the Wt counterparts (Fig. 4b). In mice receiving saline injection, there was no significant difference in the protein levels of GFAP between Wt (100 ± 11% and 100 ± 18% for cortex and hippocampus, respectively, *n* = 5) and sEH^−/−^ mice (84 ± 13% and 88 ± 15% of Wt control with saline for cortex and hippocampus, respectively, *n* = 4). An increased hippocampal iNOS at protein levels was also found in sEH^−/−^ mice that received LPS (146 ± 7% of Wt control with LPS, *p* < 0.05), whereas cortex iNOS was slightly increased.

**Fig. 5:**
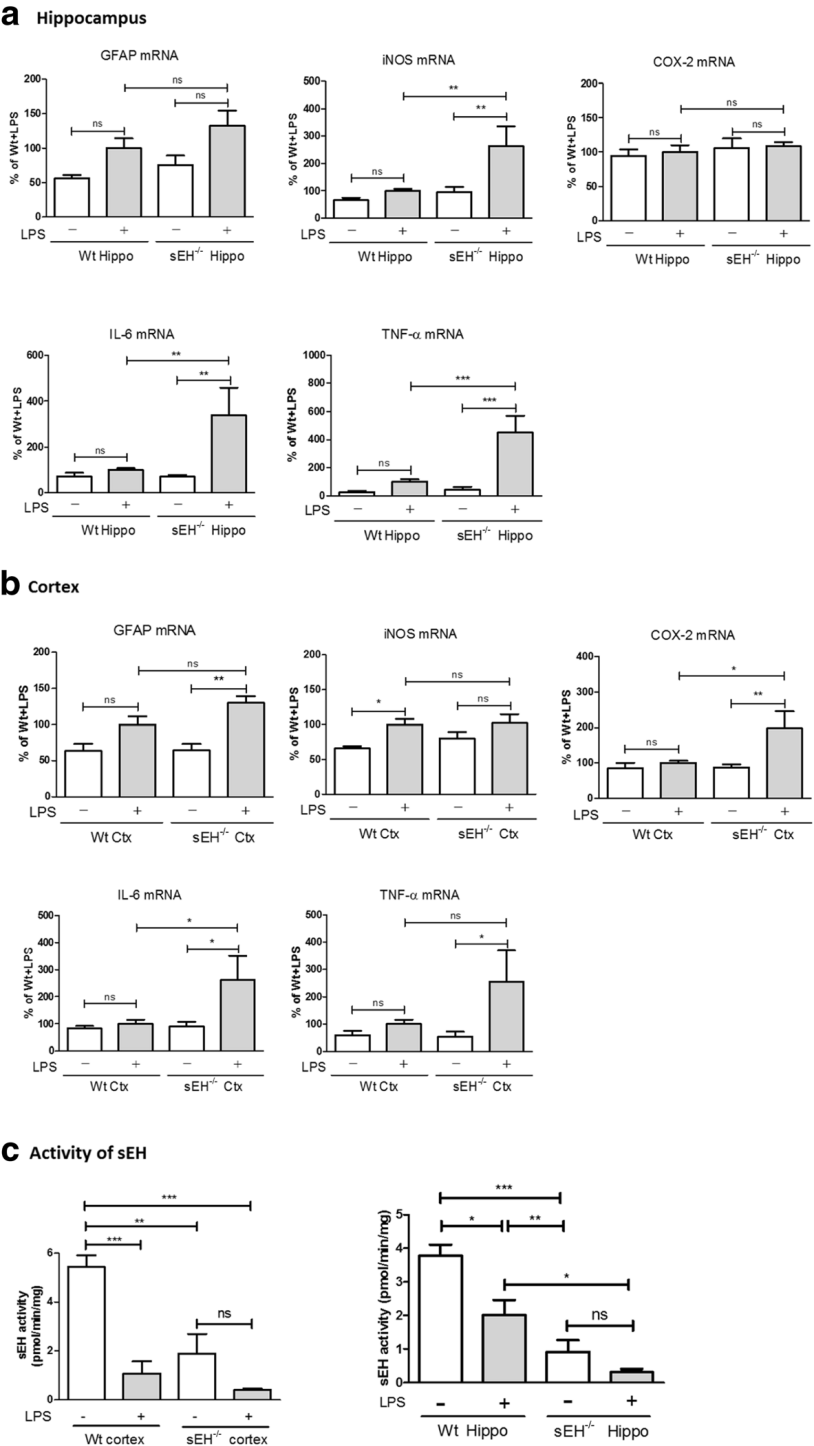
The LPS-induced mRNA expressions of pro-inflammatory markers were enhanced in the brains of sEH^−/−^ mice. After intraperitoneal injections of LPS, the mRNA levels of GFAP and pro-inflammatory markers, including iNOS, COX-2, IL-6, and TNFα were evaluated in the hippocampus (**a**) and cortex (**b**) by qPCR. The data indicate that the basal levels of mRNA for all markers were comparable between wt (*n* = 9) and sEH^−/−^ (*n* = 8) mice that received saline treatments. LPS treatments in Wt (*n* = 10) slightly increased the mRNA expression levels of GFAP and the pro-inflammatory markers in the examined brain regions except for iNOS in the cortex (*p* < 0.05), whereas LPS treatments in sEH^−/−^ mice (*n* = 6) significantly increased iNOS (*p* < 0.01), IL-6 (*p* < 0.01), and TNFα (*p* < 0.001) in the hippocampus and GFAP (*p* < 0.01), COX-2(*p* < 0.05), IL-6 (*p* < 0.05), and TNFα (*p* < 0.05) in the cortex. Compared to Wt control with LPS, the mRNA levels of iNOS (*p* < 0.01), IL-6 (*p* < 0.01), and TNFα (*p* < 0.001) in the hippocampus were further enhanced in sEH^−/−^ mice that received LPS. Similarly, significant increases of COX-2 (*p* < 0.05) and IL-6 (*p* < 0.05) in the cortex of sEH^−/−^ mice with LPS were found. These data suggest that LPS treatments may result in higher mRNA expressions of some pro-inflammatory markers in the brain of sEH^−/−^ mice than those in Wt. **c** The activity of sEH in the cortex and the hippocampus was lower in sEH^−/−^ mice (*n* = 4, *p* < 0.001) than in wt mice (*n* = 5). A significant suppression of sEH activity was found in the hippocampus (*p* < 0.05) and cortex (*p* < 0.001) of Wt mice with LPS (*n* = 6). The activity of sEH in sEH^−/−^ mice with LPS (*n* = 5) was slightly reduced, but not significant. Data are presented as the mean ± SEM. One-way ANOVA and Bonferroni multiple comparison test were performed. *p < 0.05; **p < 0.01; ***p < 0.001.

The extent of astrocyte activation was then measured by the expressions at mRNA levels for GFAP and pro-inflammatory markers, including iNOS, COX-2, IL-6, and TNFα in the hippocampus and cortex. Data indicate that intraperitoneal injections of LPS slightly elevated those genes as compared to saline controls, some of which were significantly increased in sEH^−/−^ mice. One explanation for the slight increase of these inflammatory markers induced by LPS might be due to the time-dependent effects of LPS on mRNA expression. As shown in Fig. 5, the LPS-treated sEH^−/−^ mice expressed higher mRNA levels of iNOS (264 ± 73% of the LPS-treated Wt control, *p* < 0.01), IL-6 (340 ± 119% of the LPS-treated Wt control, *p* < 0.01), and TNFα (450 ± 121% of the LPS-treated Wt control, *p* < 0.001) in the hippocampus than the LPS-treated Wt mice. However, mRNA levels of GFAP and COX-2 in the hippocampus were slightly increased in the LPS-treated sEH^−/−^ mice as compared to the LPS-treated wt mice. In the cortex, the LPS-treated sEH^−/−^ mice expressed higher mRNA levels of COX-2 (198 ± 48% of the LPS-treated Wt control, *p* < 0.05) and IL-6 (263 ± 89% of the LPS-treated Wt control, *p* < 0.05) than the LPS-treated wt mice, whereas mRNA levels of GFAP, iNOS, and TNFα were slightly increased. These results support the findings of our in vitro study, suggesting that sEH may function as a suppressor in the regulation of the LPS-activated immune response in astrocytes, while the genetic deletion of sEH may exacerbate astrocyte activation and the associated immune response. However, the levels of GFAP and the pro-inflammatory markers at the basal conditions were not affected by the genetic deletion of sEH. These findings suggest that the functional effects of sEH on the regulation of the astrocytic immune response might be associated with the activation status, and it is conceivable to speculate that the LPS-activated signaling pathways may be involved in the regulatory function of sEH during astrocyte activation. In line with this speculation, our data indeed showed that sEH activity in the hippocampus and cortex was significantly suppressed in the brains of mice that received intraperitoneal injections of LPS (Fig. 5c). These findings also echo our data as described above that mRNA levels of sEH were suppressed in primary astrocytes treated with LPS, although the reduction of sEH by acute activation by LPS is contrary to the increase of astrocytic sEH in aged APP/PS1 mice with chronic astrogliosis.

### Regulation of astrocyte activation by sEH involves the suppression of STAT3 phosphorylation

**Fig. 6:**
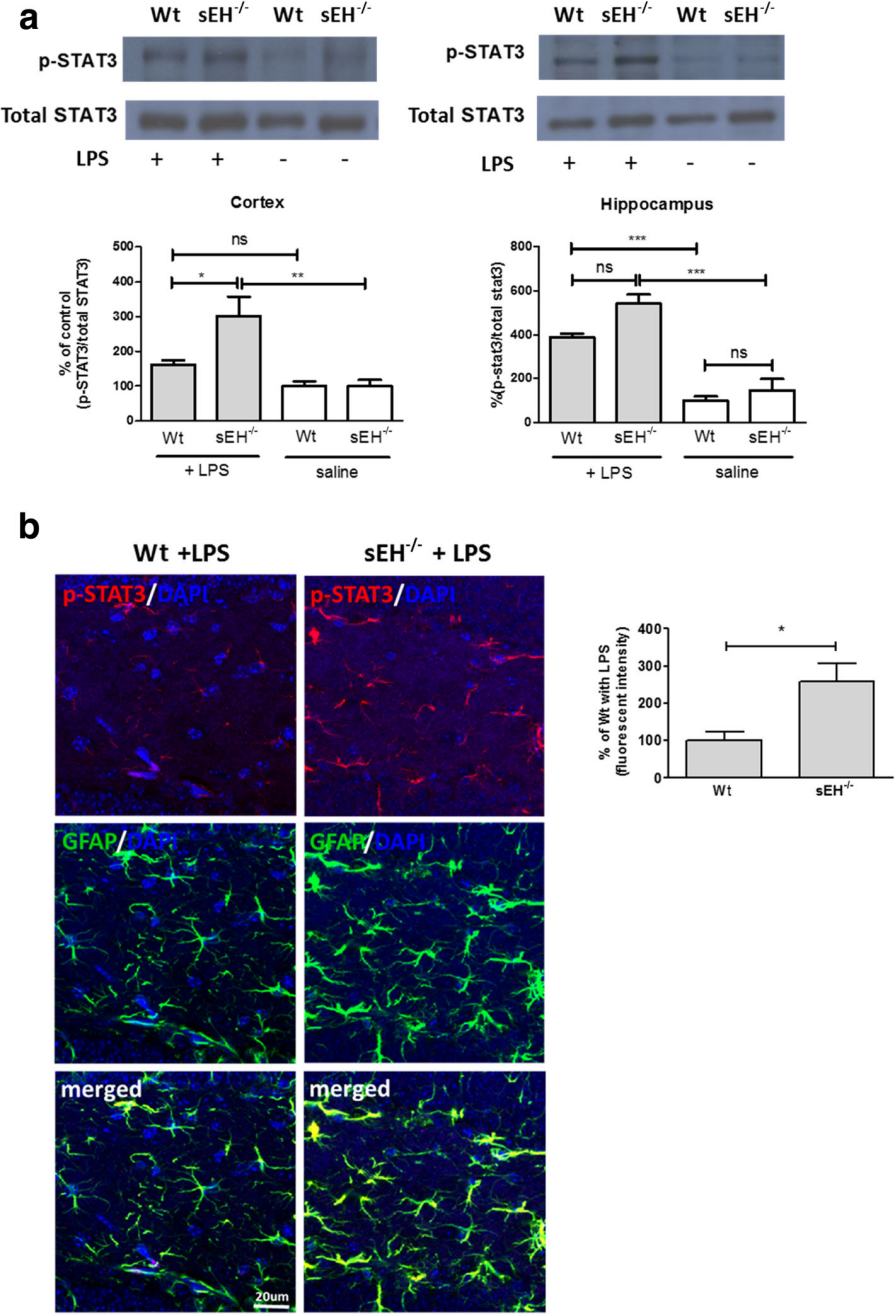
STAT3 phosphorylation was upregulated in activated astrocytes in sEH^−/−^ mice. Western blot analysis showed that the intraperitoneal injection of LPS significantly upregulated the phosphorylation of STAT3 in the hippocampus of Wt mouse brains (*n* = 4, *p* < 0.001), and this upregulation was also found in the cortex (*p* < 0.01) and the hippocampus (*p* < 0.001) of sEH^−/−^ mice (*n* = 4) (**a**). Intriguingly, LPS-induced phosphorylation of STAT3 was higher in the cortex of sEH^−/−^ mice (*p* < 0.05) than in that of Wt with LPS (*n* = 4). Although the phosphorylation of STAT3 in the hippocampus of sEH^−/−^ mice was slightly upregulated, confocal imaging showed that the phosphorylation of STAT3 (p-STAT3) was significantly higher (*p* < 0.05) in the hilus of the hippocampus of sEH^−/−^ mice that received LPS than in Wt counterparts (**b**). A complete co-localization between phosphorylated STAT3 and GFAP is demonstrated by merged images. p-STAT3 was detected by an antibody against STAT3 phosphorylated at tyrosine 705 (pTyr705). DAPI was used to label nuclei. One-way ANOVA and Bonferroni multiple comparison test were performed (**a**) and a two-tailed independent Student’s *t*-test was performed (**b**). *p < 0.05, **p < 0.01, ***p < 0.001

To explore the signaling pathways that could be attributed to the negative regulatory role of sEH in the LPS-induced astrocytic immune response, we examined the activation of nuclear factor kappa B (NFkB), P38, and janus kinase 2 (JAK2)/STAT3 in sEH^−/−^ and Wt mouse brains. The data from the Western blot analysis show that intraperitoneal injections of LPS upregulated the phosphorylation of STAT3 in mouse brains (Fig. 6a), but not JAK2, NFkB, or P38 (data not shown). Intriguingly, the phosphorylation of STAT3 was significantly higher in the cortex of sEH^−/−^ mice that received LPS (302 ± 56% of Wt control, *p* < 0.05) than in Wt counterparts (161 ± 13% of Wt control). Although a slight elevation was found in the hippocampus of sEH^−/−^ mice (541 ± 42% of Wt control) compared to the control that received LPS (387 ± 19% of Wt control), phosphorylation of STAT3 in the hippocampus of LPS-treated sEH^−/−^ mice was significantly increased (*p* < 0.05) in the hilus of the dentate gyrus as demonstrated by confocal imaging with quantification (Fig. 6b). The results show that sEH^−/−^ mice that received intraperitoneal injection of LPS showed higher levels of STAT3 phosphorylation than Wt counterparts, whereas the immunoreactivity of phosphorylated STAT3 was completely co-localized within GFAP-positive astrocytes. Based on these findings, we speculate that sEH may function as a negative regulator of astrocyte activation involving the suppression of STAT3 phosphorylation.

**Fig. 7:**
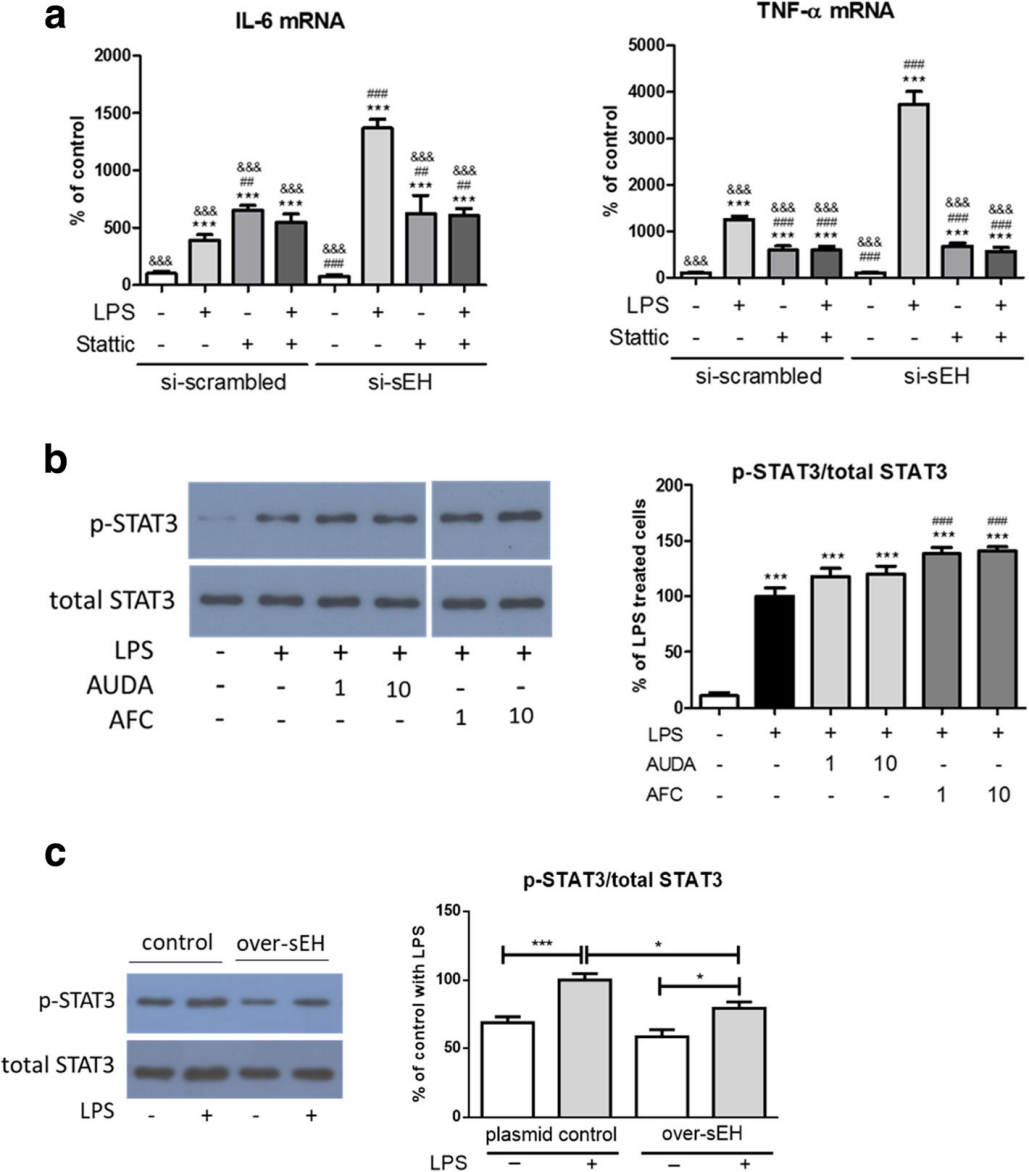
**a**–**c**The increase of LPS-induced pro-inflammatory markers in si-sEH astrocytes was suppressed by inhibition of STAT3 phosphorylation. The inhibition of STAT3 phosphorylation in LPS-activated astrocytes by the pretreatment of a STAT3 inhibitor, stattic, (10 μM) attenuated the enhanced mRNA expression levels of IL-6 and TNFα resulting from si-sEH, as measured by qPCR (*n* = 4, *p* < 0.001 for both IL-6 and TNFα). Stattic significantly suppressed LPS-induced TNFα expression in the scrambled control (*p* < 0.001) while slightly increasing LPS-induced IL-6 expression. Of note, stattic increased the mRNA expression levels for IL-6 (*p* < 0.001) and TNFα (*p* < 0.001) in astrocytes in basal conditions. ***p < 0.001, compared to si-sEH control; ^##^*p* < 0.01, ^###^*p* < 0.001, compared to si-sEH with LPS; ^&&&^*p* < 0.001, compared to si-sEH with LPS. According to the data from Western blot analysis, STAT3 phosphorylation was upregulated in LPS-activated astrocytes (*n* = 8, *p* < 0.001) and was further enhanced by pretreatments with AFC, an sEH phosphatase activity inhibitor, (138 ± 6 and 141 ± 4% of LPS-activated astrocytes for doses at 1 and 10 μM, respectively, *p* < 0.001), but not by pretreatments with AUDA, an sEH hydrolase activity inhibitor, (118 ± 7 and 120 ± 8% of LPS-activated astrocytes for doses at 1 and 10 μM, respectively) (**b**). All lanes in the representative blot images of the same target protein were obtained from the same blot membrane. ***p < 0.001, compared to control; ^###^*p* < 0.001, compared to control with LPS. In Fig. 7c, LPS-induced STAT3 phosphorylation was reduced in astrocytes overexpressing sEH (59 ± 5% of the control with LPS, n = 10, *p* < 0.05) as compared to that in plasmid control with LPS (100 ± 5). *p < 0.05, ***p < 0.001. Data are presented as the mean ± SEM. One-way ANOVA and Bonferroni multiple comparison test were performed

We then examined the effects of a pharmacological blockade of STAT3 activity on the expression levels of pro-inflammatory markers in primary astrocytes treated with si-sEH. Similar to what was observed in Fig. 3, the levels of LPS-induced TNFα (3736 ± 136% of the scrambled control, *p* < 0.001) and IL-6 (1371 ± 38% of the scrambled control, *p* < 0.001) mRNA were higher in si-sEH astrocytes than those in the scrambled control astrocytes (1258 ± 33% and 392 ± 24% of the scrambled control for TNFα and IL-6, respectively) (Fig. 7a). The inhibition of STAT3 phosphorylation by the pre-treatment of stattic, a STAT3 inhibitor, significantly attenuated the LPS-induction of TNFα mRNA in both si-sEH astrocytes and the scrambled controls. Intriguingly, the inhibition of STAT3 phosphorylation suppressed the LPS-induction of IL-6 mRNA in si-sEH astrocytes, but not in the scrambled controls. These data suggest that, in the presence of sEH, the LPS-activated upregulation of TNFα mRNA in astrocytes may act at least partly through a mechanism involving STAT3 phosphorylation, whereas induction of IL-6 mRNA by LPS may not require STAT3 phosphorylation. Importantly, in the absence of sEH, further upregulation of TNFα and IL-6 mRNA may require STAT3 phosphorylation. Therefore, sEH may function as a negative regulator of astrocyte activation and STAT3 phosphorylation may be involved in this regulatory process. Intriguingly, treatments with stattic alone in the scrambled control astrocytes promoted the expressions of these pro-inflammatory cytokines, suggesting that STAT3 activity may exert multiple functions in the regulation of astrocyte immune response.

It has been reported that sEH possesses phosphatase and hydrolase activities. We then investigated whether the regulation of STAT3 phosphorylation during astrocyte activation was differentially affected by these two functional domains of sEH. Pretreatments of AUDA and AFC were applied, to inhibit the sEH hydrolase activity and phosphatase activity, respectively, in LPS-activated astrocytes. In Fig. 7b, the data show that STAT3 phosphorylation was upregulated in LPS-activated astrocytes and was further enhanced by pretreatments of AFC (138 ± 6% and 141 ± 4% of the control with LPS for 1 μM and 10 μM of AFC, respectively) but not by pretreatments of AUDA. In contrast, LPS-induced STAT3 phosphorylation was reduced in astrocytes overexpressing sEH (59 ± 5% of the control with LPS, *p* < 0.05) compared to the control with LPS (100 ± 5%) (Fig. 7c). These data suggest that the phosphatase, but not the hydrolase, activity of sEH may contribute to the negative regulation of STAT3 phosphorylation.

## Discussion

Astrocyte activation is an important pathological feature in AD and many other neurodegenerative diseases [34, 35]. Understanding the underlying regulatory mechanism may confer a molecular basis for the development disease therapies. Our data show that the expression of sEH was positively associated with the progression of astrocyte activation in APP/PS1 transgenic mice, while sEH immunoreactivity was barely detectable in resting astrocytes with a quiescent phenotype. Importantly, we demonstrated, for the first time, that sEH plays a role in the negative regulation of LPS-induced astrocyte immunity, as evidenced by data from in vivo and in vitro models. Data suggest that the genetic deletion of sEH, either sEH^−/−^ or si-sEH, enhanced the production of LPS-induced pro-inflammatory markers, whereas the overexpression of sEH ameliorated the immune response. However, the involvement of microglial activation in the enhanced pro-inflammatory markers found in sEH^−/−^ mice cannot be excluded due to the constant interactions between microglia and astrocytes in the brain. Furthermore, our findings identify sEH as a novel inhibitory effector in the regulation of STAT3 activity and demonstrate the critical role of STAT3 in astrocyte activation. Based on these findings, we propose that the immune-suppressing function of sEH involving STAT3 inhibition may play an important role in the regulation of astrocyte activation.

### The expression of sEH in astrocyte activation in AD pathogenesis

The number of GFAP-positive astrocytes near Aβ plaques increases as AD progresses. Similarly, our data show that astrocytic sEH immunoreactivity increased in parallel with the extent of astrogliosis and was limited to the activated astrocytes in proximity to Aβ plaques. However, astrocytes in the brain of AD mice appear to express different levels of sEH, and less activated astrocytes that were close to Aβ plaques expressed little or no sEH, as shown in the abovementioned results. One possible explanation for these findings is that the different levels of sEH expression in response to an inflammatory microenvironment may be due to the heterogeneity of astrocytes [36]. Indeed, it has been reported that subtypes of activated astrocytes, with distinct functionality in the diseased brain, exist [15]. Alternatively, the increase of sEH expression in astrocytes may be associated with the progression of chronic disease status, and Aβ exposure per se may not necessarily instigate the expression of sEH in activated astrocytes. Given that various soluble Aβ species, including Aβ oligomers, are widespread in the brain, the exposure of activated astrocytes to Aβ is inevitable. Moreover, as demonstrated in our data, acute stimulation by LPS reduced, rather than increased, sEH in mice and primary astrocytes. One explanation is that the unspecified LPS-activated signaling might be involved in the acute effect of LPS on reducing the expression of sEH, whereas chronic activation leading to severe astrogliosis triggers upregulation of astrocytic sEH to counteract the inflammatory state. Therefore, the more the astrocytes are in an advanced and chronic activation, the more sEH is expressed. This speculation echoes our observation showing that intensive sEH immunoreactivity was commonly found within the enlarged processes of highly activated astrocytes in aged APP/PS1 mice. In contrast, activated astrocytes in younger APP/PS1 mice showed less sEH immunoreactivity. Whether the extent of sEH expression in activated astrocytes could be indicative of disease progression and perhaps serves as a biomarker for AD remains to be explored.

Nevertheless, the fact that levels of sEH modulate immune response in activated astrocytes may have significant impacts on AD pathogenesis. We speculate that high sEH-expressing astrocytes could be a subtype of astrocytes that are attempting to cope with inflammation because the expression of sEH could be anti-inflammatory in activated astrocytes. Therefore, it is conceivable to propose that activated astrocytes with high expression levels of sEH could be beneficial for counteracting the over-activation of astrocyte. However, sEH-overexpressing astrocytes, on the other hand, may reduce their beneficial functions of immune response against the disease progression. In fact, the beneficial functions of activated astrocytes have been recently reported, showing that Aβ plaques with little or no activated astrocytes were associated with dementia status in AD patients [37, 38]. Therefore, the possible adverse effects of sEH overexpression on over-reducing astrocytic functions remained to be elucidated.

### Regulation of astrocyte activation by sEH involving STAT3 inhibition

Our study support the notion that STAT3 activation can be pro-inflammatory and is involved in triggering the expression of pro-inflammatory markers in activated astrocytes [10]. In addition, we further revealed a novel function of sEH in the regulation of STAT3 activation in activated astrocytes. Based on our findings, the genetic deletion or pharmacological blockade of sEH led to an increase in LPS-induced STAT3 phosphorylation in activated astrocytes, which can be significantly attenuated by the inhibition of STAT3 using stattic in LPS-activated astrocytes. These data suggest that the STAT3-mediated astrocyte activation plays an important role in the regulation of LPS-activated astrocyte activation, whereas the pro-inflammatory properties of STAT3 are likely to be at least partly negatively regulated by sEH. In addition to its pro-inflammatory properties, many studies have reported that STAT3 activity can be anti-inflammatory and might promote the expressions of many anti-inflammatory genes, resulting in the indirect inhibition of pro-inflammatory gene expression in dendritic cells or macrophages [39, 40]. Indeed, our data show that stattic alone triggered the expression of TNFα and IL-6 in astrocytes at resting condition. It is conceivable to speculate that the upregulation of the pro-inflammatory cytokines by stattic in astrocytes at basal levels might result from the blockade of the anti-inflammatory mode of STAT3. However, the possibility that effects of stattic on the expression of pro-inflammatory cytokines are independent of STAT3 activation cannot be excluded. Taken together, the sEH-mediated STAT3 inhibition may play multiple roles in the regulation of astrocyte activation. Further research is needed to clarify how sEH interacts with STAT3 and how the sEH-mediated STAT3 inhibition contributes to the pathogenesis of AD.

## Conclusions

In conclusion, we demonstrated, for the first time, that the deletion of sEH in astrocytes was pro-inflammatory, whereas the overexpression of sEH suppressed the immune response. The underlying mechanism may be at least partly attributed to the regulation of STAT3 activation. The sEH-mediated STAT3 inhibition in a cellular context suggests that astrocytic sEH may play an important role in the regulatory machinery of pro-inflammatory response in activated astrocytes during the progression of the brain diseases. Revealing the underlying mechanisms through which sEH orchestrates STAT3 activity and developing means to manipulate astrocytic sEH expression could facilitate the future development of treatment for brain diseases.

## Additional file

Additional file 1: **Figure S1.** The expression levels of sEH by genetic manipulations in astrocytes. The expression levels of sEH in primary astrocytes derived from Wt mice were genetically manipulated by using commercially available reagents for the RNA silencing of sEH (si-sEH) and the overexpression of sEH (over-sEH). The levels of sEH mRNA in si-sEH and over-sEH astrocytes were 28 ± 3% (*n* = 9) and 5.8 ± 1.2 × 10^6^% (*n* = 6) of the control, respectively, as measured by qPCR (Additional file 1: Figure S1A, B). The mRNA levels of sEH in non-transfected primary astrocytes appeared to be downregulated by LPS treatments (38 ± 5% of the control, *n* = 7, *p* < 0.001) (Additional file 1: Figure S1C). Data are presented as the mean ± SEM. One-way ANOVA and Bonferroni multiple comparison test were performed for (A) and (B), whereas a two-tailed independent Student’s *t*-test was performed for (C). ***p < 0.001. **Figure S2.** Pro-inflammatory markers in primary astrocytes from sEH^−/−^. Protein levels of iNOS and COX-2 were significantly increased by LPS treatments in primary astrocytes derived from either Wt or sEH^−/−^ mice. However, the LPS-induced expressions of these pro-inflammatory markers were not affected by sEH^−/−^. Data are presented as the mean ± SEM. One-way ANOVA and Bonferroni multiple comparison test were performed. *p < 0.05, **p < 0.01, ***p < 0.001. (DOCX 99 kb)

## Data Availability

Not applicable

## Abbreviations

AD: Alzheimer’s disease
AFC: N-acetyl-S-farnesyl-L-cysteine
APP/PS1: APPswe/PSEN1dE9, an Alzheimer’s disease mouse model
AUDA: 12-(3-adamantan-1-yl-ureido)-dodecanoic acid
Aβ: β-amyloid
COX-2: Cyclooxygenase 2
ECL: Electrochemiluminescence
EETs: Epoxyeicosatrienoic acids
GFAP: Glial fibrillary acidic protein
HETEs: Hydroxyeicosatetraenoic acids
IL-6: Interleukin-6
iNOS: Inducible nitric oxide
JAK2: Janus kinase 2
LPS: Lipopolysaccharides
NFkB: Nuclear factor kappa B
NO: Nitric oxide
over-sEH: Overexpression of sEH
sEH: Soluble epoxide hydrolase
sEH^−/−^: SEH knockout
si-sEH: Silencing of sEH
STAT3: Signal transducer and activator of transcription 3
TNFα: Tumor necrosis factor α
Wt: Wild-type
