# Supplementary figures and images for: Soluble epoxide hydrolase modulates immune responses in activated astrocytes involving regulation of STAT3 activity
Source: J Neuroinflammation. 2019 Jun 8;16:123. doi: 10.1186/s12974-019-1508-2 (PMC6555999; doi:10.1186/s12974-019-1508-2)

**
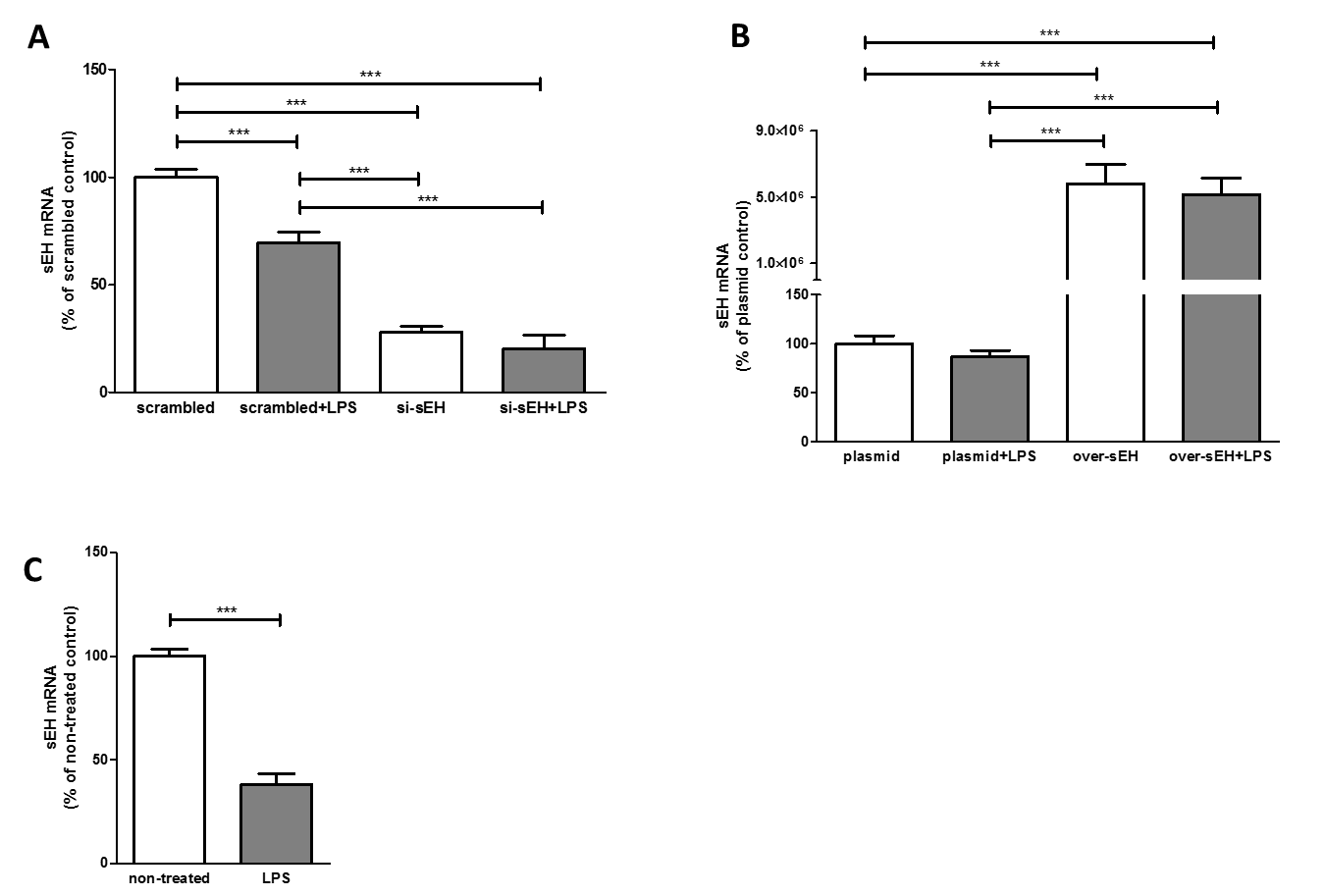
**

**Supplementary Figure 1.**

**
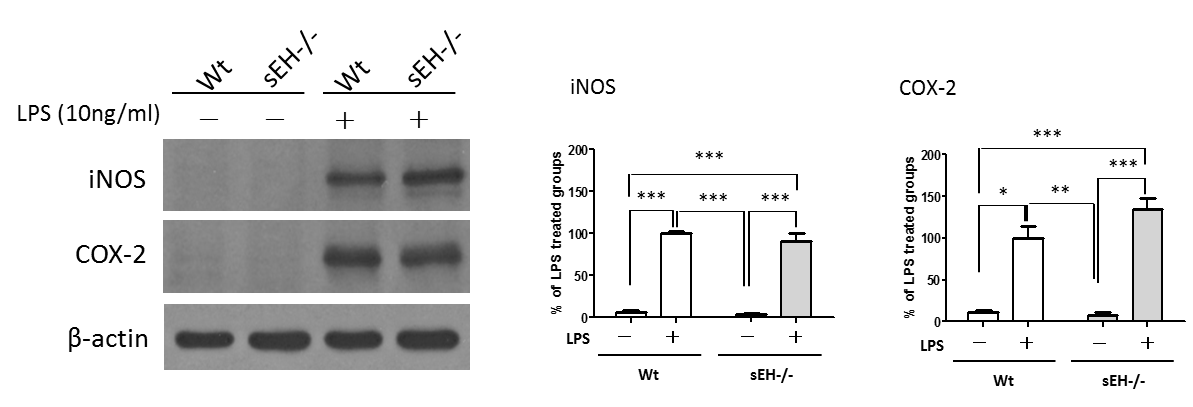
**

**Supplementary Figure 2.**

Supplement: Supplementary file 1 — Figure S1. The expression levels of sEH by genetic manipulations in astrocytes. The expression levels of sEH in primary astrocytes derived from Wt mice were genetically manipulated by using commercially available reagents for the RNA silencing of sEH (si-sEH) and the overexpression of sEH (over-sEH). The levels of sEH mRNA in si-sEH and over-sEH astrocytes were 28 ± 3% (n = 9) and 5.8 ± 1.2 × 106% (n = 6) of the control, respectively, as measured by qPCR (Additional file 1: Figure S1A, B). The mRNA levels of sEH in non-transfected primary astrocytes appeared to be downregulated by LPS treatments (38 ± 5% of the control, n = 7, p < 0.001) (Additional file 1: Figure S1C). Data are presented as the mean ± SEM. One-way ANOVA and Bonferroni multiple comparison test were performed for (A) and (B), whereas a two-tailed independent Student’s t-test was performed for (C). ***p < 0.001. Figure S2. Pro-inflammatory markers in primary astrocytes from sEH−/−. Protein levels of iNOS and COX-2 were significantly increased by LPS treatments in primary astrocytes derived from either Wt or sEH−/− mice. However, the LPS-induced expressions of these pro-inflammatory markers were not affected by sEH−/−. Data are presented as the mean ± SEM. One-way ANOVA and Bonferroni multiple comparison test were performed. *p < 0.05, **p < 0.01, ***p < 0.001. (DOCX 99 kb) [file 12974_2019_1508_MOESM1_ESM.docx]
